# Cognitive abnormalities in Becker muscular dystrophy: a mysterious link between dystrophin deficiency and executive functions

**DOI:** 10.1007/s10072-023-07169-x

**Published:** 2023-11-15

**Authors:** Laura Pezzoni, Roberta Brusa, Teresa Difonzo, Francesca Magri, Daniele Velardo, Stefania Corti, Giacomo Pietro Comi, Maria Cristina Saetti

**Affiliations:** 1 Foundation IRCCS Ca’ Granda Ospedale Maggiore Policlinico, Neurology Unit, Milan, Italy; 2 ASST Ovest Milanese, Ospedale Di Legnano, Neurology Unit, Legnano, Milan, Italy; 3 Foundation IRCCS Ca’ Granda Ospedale Maggiore Policlinico, Neuromuscular and Rare Diseases Unit, Milan, Italy; 4 Department of Pathophysiology and Transplants, Dino Ferrari Center, University of Milan, Milan, Italy

**Keywords:** Becker muscular dystrophy, Cognition, Neuropsychological tests, Executive functions

## Abstract

**Background:**

Distrophinopathies are a heterogeneous group of neuromuscular disorders due to mutations in the *DMD* gene. Different isoforms of dystrophin are also expressed in the cerebral cortex and Purkinje cells. Despite cognitive abnormalities in Duchenne muscular dystrophy subjects that have been described in the literature, little is known about a comprehensive cognitive profile in Becker muscular dystrophy patients.

**Aim:**

The aim of this study was to assess cognitive functioning in Becker muscular dystrophy patients by using an extensive neuropsychological battery. Our hypothesis is that the most impaired functions are the highly intentional and conscious ones, such as working memory functions, which require a prolonged state of cellular activation.

**Methods:**

We performed an extensive neuropsychological assessment on 28 Becker muscular dystrophy patients from 18 to 65 years old. As control subjects, we selected 20 patients with limb-girdle muscular dystrophy, whose clinical picture was similar except for cognitive integrity. The evaluation, although extended to all areas, was focused on prefrontal control skills, with a distinction between inhibitory processes of selective attention and activating processes of working memory.

**Results and conclusions:**

Significant underperformances were found exclusively in the Dual Task and PASAT tests, to demonstrate a selective impairment of working memory that, while not causing intellectual disability, reduces the intellectual potential of patients with Becker muscular dystrophy.

## Introduction

Dystrophinopathies are related to the absence (Duchenne muscular dystrophy, DMD) or to the partial deficiency (Becker muscular dystrophy, BMD) of the dystrophin protein, encoded by the *DMD* gene on chromosome X. Although dystrophin is mainly expressed in the skeletal muscle, different isoforms are also expressed in other tissues, including the brain. The massive gene of dystrophin contains in fact a set of tightly regulated promoters that generate eight cell-specific protein isoforms, which all share the same C-terminal domain but start from different N-terminal domains [1]. In neural cells, no less than five isoforms of dystrophin are expressed: two full-length isoforms, cerebellar dystrophin and cortical dystrophin, and three short-form isoforms: Dp140, Dp116, and Dp71, which are the most abundant in the brain [2]. The function of all dystrophin isoforms in the brain is not entirely understood yet, but they appear to be involved in myelination during neural development [3], synaptic modulation [4], and neuronal differentiation through neuritis growth [5] as well as in cellular energy metabolism [6]. Their complete or partial loss in DMD and BMD seems to underlie the great variability of cognitive deficits observed in these individuals. Many studies in fact show a correlation between the risk of cognitive impairment in both DMD and BMD and cumulative loss of functional isoforms expressed in the brain, determined by mutation position along the gene [2, 7]. Cognitive abnormalities associated with DMD are well described in literature: the drastic loss of brain dystrophins is likely to cause a global alteration in neural and cortical development [8], resulting in compromised intellectual functioning (medium intellective quotient (IQ) according to the Wechsler scale one standard deviation lower than the general population and 19–35% of intellectual disability) [9], deficits in working memory, executive functions, reading and writing [10], and neuropsychiatric disorders [11]. Much less is known regarding a comprehensive cognitive profile in BMD. A recent systematic review of literature by Ferrero and Rossi [12] highlights the scarcity of studies focusing on cognitive aspects of BMD and how most papers are primarily interested in the evaluation of global QI over the characterization of specific cognitive defects in these subjects. The consensus is that, despite an increased risk of intellectual disability (7–25%) in children and adults affected by BMD, average QI does not differ from the general population. Only two studies to date aim to analyze the cognitive profile of BMD individuals, the works of Young et al. [13] and the pilot study by Biagi et al. [14]. Both works show a deficit in executive functions in BMD individuals, especially in working memory. Nonetheless, the scant literature available presents some limitations. First of all, studies often include a population of both children and adults [12], reaching conflicting conclusions. Moreover, deficits in executive functions and working memory were inferred from verbal subtests of WISC-III and not using specific tests assaying executive functions [13, 14]. Finally, the studies confront the intellectual functions of children whose motor impairment and general frailty affect social relationships and psychological equilibrium with those of children in normal living conditions.

The present study arises from the hypothesis that the BMD neuropsychological profile is characterized by a preserved global intellectual functioning with a deficit in executive functions and especially working memory. Contrary to what happens in DMD, in fact, individuals affected by BMD have in most cases enough brain dystrophin to ensure a physiological cortical development and therefore a preserved global intellectual functioning (IQ). Nonetheless, given its role in cellular energy metabolism and membrane stability, dystrophin deficiency could disrupt neural communication, taking a heavier toll on areas that establish numerous connections throughout the brain and that support cognitive functions with high energy impact, namely the prefrontal cortex and executive functions. “Executive” or “control” functions (EF) are a group of higher-level functions that, influencing lower-level sensory and motor processes as well as other cognitive functions, orchestrate our thoughts and behavior in accordance with internal goals [15]. All control functions are highly intentional, conscious, and complex and require a prolonged cell activation state [15]. However, selective attention arises from inhibitory processes (inhibition of stimuli, thoughts, or actions that interfere with the objective), whereas working memory is mainly based on activating pathways possibly requiring more energy, and thus, it may be more affected by dystrophin deficiency in BMD. Working memory (WM) is the ability to process information temporarily kept in consciousness, thanks to the short-term memory “reservoir,” to achieve a particular goal [16]. Every time a piece of information is used for any cognitive task (e.g., reviewed or employed to make a decision), we are exercising WM, whose neural substrates include the cerebellum and especially the dorsolateral prefrontal cortex (DLPFC) [17].

To exert cognitive control, EF arise from circuits that involve the brain globally, but the prefrontal cortex (PFC) is considered a key neural substrate for all of them [15]. It establishes connections with sensory and high-order associative areas, motor cortexes, and subcortical structures [15], integrating multiple stimuli for an accurate representation of inner needs and environmental context and requests [18, 19], and influencing the subordinate areas by inhibition and activation of neural pathways [20]. Within PFC, the orbito-mesial prefrontal cortex (OFC) is specifically involved in value-based decision-making [21], while the dorsolateral prefrontal cortex (DLPFC) finally receives and processes information from all the other prefrontal areas to finalize behavioral choices, sending outputs to premotor and motor areas [22]. It guarantees the automation and velocity of decision-making [23], and it is superordinate of all the prefrontal areas standing at the very vertex of the hierarchy of cognition. Moreover, it is specialized in the control, selection, and manipulation of information within working memory [17]. Extremely complex signal integration processes take place at this level, requiring considerable metabolic input, hence making in our opinion DLPFC in particular—and more broadly PFC—extremely sensitive to a dystrophin deficiency. Observationally, in the neuropsychological examination of BMD subjects, we expect to find a selective deficit of executive functions and specifically of working memory, despite a normal intellectual functioning. The aim of this study is therefore to assess cognitive functioning in BMD subjects using an extensive neuropsychological battery, focusing especially on executive functions.

## Methods

### Cohort selection

Twenty-eight male adult patients with Becker muscular dystrophy (BMD) and 19 adult male and female patients with limb-girdle muscular dystrophy (LGMD) were enrolled in this study.

BMD patients were recruited during a 9-month period from patients with phenotypic, bioptic, and immunohistochemical characteristics and molecular diagnosis of Becker muscular dystrophy afferent to the Neuromuscular Diseases Center, Neurology U.O.C. of the “Policlinico Maggiore” Hospital, in Milan. Patients were excluded in case of suspected clinical cognitive decline, history of any other significant neurological or psychiatric disorder, non-neurological comorbidities that could potentially compromise neuropsychological testing results, and use of psychotropic drugs.

Genetic diagnosis of dystrophinopathy was carried out by MLPA (multiple ligation-depended probe amplification) and, if negative, by direct sequencing of the *DMD* gene. Most patients (26/28) carry a deletion of one or more exons in the dystrophin gene, the other 2 patients carry a microdeletion (c.676–678del). Among patients with exon deletion, exon 45 is involved in 18 subjects: 13 subjects carry a 45–47 deletion, three subjects a 45–48 deletion, one subject a 45–49 deletion, and one subject a 45–53 deletion. In the remaining cases, we have exon 3–4 deletion (3 subjects), exon 3–6 deletion (2 subjects, siblings), exon 3–7 deletion (1 subject), and exon 12–19 deletion (2 subjects). Of all the patients, only one subject carries the 45–53 deletion, which includes exons 51 to 53 and is predicted to disrupt Dp140. No further patients carry mutations in the distal portion of the *DMD* gene, known in the literature to be associated with higher rates of intellectual disability and cognitive abnormalities [7].

LGDM patients were enrolled as control subjects, to exclude that an environmental bias, related to the physical and social limitations imposed by the motor impairment, interfered with the comparisons. LGMDs are a complex and heterogeneous group of disorders, whose clinical expression is similar to that of dystrophinopathies, with which they enter into differential diagnosis. They are caused by the mutation of proteins essential for muscle and heart fibers, without CNS involvement. LGMD patients were recruited too during a 9-month period among patients of the Neuromuscular Diseases Centre, Neurology U.O.C. of the “Policlinico Maggiore” Hospital, in Milan. Inclusion criteria were clinical findings of limb-girdle muscular dystrophy, dystrophic changes at muscle biopsy, and genetic mutation indicative of LGMD. Exclusion criteria are the same as above for BMD patients; moreover, we have excluded patients whose LGMD was due to POMT1 and POMT2 mutations (LGMD R11 and LGMD R14 dystrophies) since they are associated in the literature with the presence of cognitive deficits [24].

Our LGMD cohort was comprehensive of three subjects affected by LGMD R2, six subjects affected by LGMD R1, two subjects affected by LGMD R12, four subjects affected by LGMD R4, two subjects affected by LGMD R5, one subject affected by LGMD R3, one subject affected by LGMD 23, and one subject affected by LGMD R9.

Informed written consent was acquired from all participants during the first evaluation, and this research was approved by the Ethics Committee of IRCCS Foundation Ca’ Granda Ospedale Maggiore Policlinico of Milan.

The clinical and demographic characteristics of the selected patients are shown in Table 1 (see Table 1). Disease severity is expressed by the MFM scale score (range 1–96) [25]. The two groups did not differ significantly in age (*t* = 1.28; df = 45; *p* < 0.002) and schooling (*t* = 1.75; df = 45; *p* = ns), disease severity (*t* = 1.30; df = 40; *p* = ns), and duration (*t* = 0.365; df = 44; *p* = ns), while they differed in gender, BMD being an x-linked disease expressed exclusively in males. We decided to include female LGMD control subjects in order not to reduce the group size. The difference was taken into account in the data analysis.

**Table 1:** Mean values (standard deviations) of demographic variables of BMD e LGMD patients compared

	BMD	LGMD	Comparison
Sex	M, 28	M, 12	
	F, 0	F, 7	
Age (years)	35.96 (11.60)	43.25 (18.70)	T = 1.28 *p* = *ns* Df = 45
Education (years)	12.00 (3.10)	11.95 (3.23)	T = 1.75 *p* = *ns* Df = 45
Disease severity (MFM)	76.00 (6.01)	70.81 (18.94)	T = 1.30 *p* = *ns* Df = 40
Disease duration (years)	25.25 (9.54)	24 (13.74)	T = 0.37 *p* = *ns* Df = 44

### Neuropsychological assessment

All patients underwent a series of standardized neuropsychological tests, including a self-reported depression questionnaire, in a single session lasting approximately 90 min. Two LGMD patients were not able to perform motor tasks because of the severity of the motor disorder in the upper limbs, and three LGMD patients could not perform verbal tasks because of severe bucco-facial impairment. All BMD patients underwent the full battery, except for one patient, not an Italian native speaker, who refused to perform the Word-pair learning task. The evaluation, although extended to all areas, was focused on prefrontal control skills, with a distinction between inhibitory processes of selective attention and activating processes of working memory.

Short-term memory and working memory are intrinsically linked; there are no specific neuropsychological tests for either function; they are always assessed together. However, a task will engage WM the more the manipulation of the elements is required [18]. The tests assaying working memory in the battery, listed in order from those that engage working memory the most to those engaging it the least, are as follows: Dual Task [26], Paced Auditory Serial Addition Test (PASAT) [27], Corsi Span-Backward, and Digit Span-Backward [28].

The battery also included tests assaying other prefrontal functions such as attention, cognitive flexibility, and deductive reasoning, such as Frontal Assessment Battery (FAB) [29], Raven’s Coloured Progressive Matrices [30], Trail Making Test (TMT) [31], Alternate Fluency Test [32].

Memory functions were tested using Digit Span-Forward and Corsi Span-Forward [28]; word-pair learning task [33] and Recurrent Faces Test [34].

Language was tested using the Boston Naming Test [35] and the Verbal Fluency Test [32]. Constructive skills were evaluated with the Drawing Copy Test [36].

Lastly, the Beck Depression Inventory-II (BDI-II) [37] was included in the battery in order to screen for affective disorders that could influence test performance.

### Statistical analyses

A linear mixed model was adopted to test the effect of the Group on the corrected scores, addressing the subject as the cluster. Normality assumptions were tested on residual, graphical (Q-Q plot), and inferential levels (Shapiro Wilk’s). The significance level was set a *α* = 0.05. For each test, we compared the frequency of BMD and LGMD pathological scores by chi-squared test. We used normative data from the literature to establish whether the scores achieved by each patient are pathological compared to the healthy population.

## Results

A significant main effect of group $(\mathrm{F}(\mathrm{19,45})=7.95;p=.048)$ was detected. Univariate tests show that BMD patients perform significantly worse than LGMD patients on the Dual Task $(\mathrm{F}(\mathrm{1,45})=10.70;p=.002)$.

Furthermore, BMD patients’ scores tend to be lower although they do not differ significantly on the PASAT $(\mathrm{F}(\mathrm{1,45})=3.21;p=.081)$ and Raven’s test $(\mathrm{F}(\mathrm{1,45})=3.02;p=.090)$.

Unexpectedly, BMD patients score better than LGMDs on the word-pair learning task $(\mathrm{F}(\mathrm{1,45})=7.37;p=.010)$. The mean test scores of BMD and LGMD patients and between-group comparisons are shown in Table 2.

**Table 2:** Mean (standard deviation) of the scores. Between-groups comparison

	BMD		LGMD		*F*	*p*
Dual task	75.42	(21.45)	94.58	(7.39)	10.69850	0.002^1^
PASAT	38.47	(11.94)	43.15	(6.55)	3.21513	0.081^2^
Corsi span-backward	4.46	(1.02)	4.82	(0.92)	2.44458	0.126
Digit span-backward	3.81	(0.87)	4.26	(1.20)	1.83425	0.184
Corsi span	5.52	(1.26)	5.47	(1.21)	0.10686	0.746
Digit span	6.01	(0.92)	5.94	(1.08)	0.13825	0.712
TMT A	37.89	(11.05)	34.56	(16.92)	0.60472	0.442
TMT B	93.79	(40.48)	102.31	(31.57)	0.26984	0.606
TMT B-A	55.57	(34.66)	61.25	(35.81)	0.17383	0.679
Phonemic fluency	36.24	(9.22)	36.82	(11.50)	0.30850	0.582
Semantic fluency	41.71	(6.20)	43.24	(9.75)	1.47614	0.232
Alternate fluency	31.67	(8.14)	31.82	(8.71)	0.07805	0.781
Composite shifting score	0.80	(0.18)	0.80	(0.14)	0.01387	0.907
FAB	15.99	(1.60)	16.16	(1.27)	0.48001	0.493
Raven’s colored PM test	30.09	(3.50)	31.68	(3.00)	3.02325	0.090^2^
Learning word pairs	14.62	(2.87)	12.06	(3.36)	7.36878	0.010^1^
Face learning	20.63	(2.69)	19.93	(2.99)	0.22016	0.642
Boston naming test	51.64	(5.26)	49.28	(5.56)	2.64831	0.112
Copy of drawings	13.28	(1.67)	12.72	(1.57)	0.15002	0.701

^1^Significant between-group comparison

^2^Tendency towards significance

The data of either pathological group did not clearly conform to a normal distribution. The scores obtained by BMD and LGMD patients on the Dual Task and the Drawing Copy Test were not normally distributed, nor were the scores obtained by BMD patients on the Corsi Span-Backward and FAB.

Comparisons between groups with nonparametric statistics (Kruskal–Wallis test) confirmed a significant difference in performance at Dual Task ${(\upchi }^{2}=12.22;p<.001)$, while BMD patients did not differ from LGMDs at all other tests.

BMD patients achieve more falls than LGMDs on Dual Task (${\upchi }^{2}=11.43;p<.005)$ and PASAT ${(\upchi }^{2}=6.00;p<.025)$. In all other tests, no significant differences emerged between groups. Frequencies of pathological scores and between-group comparisons are shown in Table 3.

**Table 3:** Frequencies of pathological scores. Between-group comparison

	BMD		LGMD		χ^2^	*p*
Dual task	14/28	(50.0%)	0/17	(0.0%)	12.300	< 0.001^1^
PASAT	7/28	(25.0%)	0/17	(0.0%)	5.030	0.025^1^
Corsi span-backward	6/28	(21.4%)	1/17	(5.9%)	1.950	0.163
Digit span-backward	9/28	(32.1%)	3/19	(15.8%)	1.590	0.207
Corsi span	6/28	(21.4%)	3/17	(17.6%)	0.094	0.758
Digit span	2/28	(7.1%)	1/19	(5.3%)	0.067	0.796
TMT A	1/28	(3.6%)	0/16	(0.0%)	0.585	0.444
TMT B	0/20	(0.0%)	0/16	(0.0%)	0.000	1.000
TMT B-A	2/28	(7.1%)	1/16	(6.3%)	0.013	0.910
Phonemic fluency	2/28	(7.1%)	1/17	(5.9%)	0.027	0.869
Semantic fluency	2/28	(7.1%)	2/17	(11.8%)	0.279	0.597
Alternate fluency	1/28	(3.6%)	0/17	(0.0%)	0.621	0.431
Composite shifting score	2/28	(7.1%)	0/17	(0.0%)	1.270	0.260
FAB	2/28	(7.1%)	1/17	(5.9%)	0.027	0.869
Raven’s Coloured PM test	0/28	(0.0%)	0/19	(0.0%)	0.000	1.000
Learning word pairs	0/28	(0.0%)	1/18	(5.6%)	1.590	0.207
Face learning	2/28	(7.1%)	3/19	(15.8%)	0.890	0.345
Boston naming test	1/28	(3.6%)	2/18	(11.1%)	1.02	0.312
Copy of drawings	1/28	(3.6%)	1/17	(5.9%)	0.133	0.715

^1^Significant between-group comparison

The two groups also do not differ in the percentage of scores indicative of depression (BMD = 10.7%, LGMD = 25%; χ2 = 1.55, *p* = ns) nor by mean score (BMD = 5.93 ± 8.00, LGMD = 8.94 ± 7.80; F(1.42) = 1.47, p = ns).

## Discussion

We elected as control subjects 19 patients affected by limb-girdle muscular dystrophy (LGMD), a heterogeneous group of congenital myopathies with different genetic basis and pathogenesis, defined by a clinical picture similar to that of BMD, except for cognitive integrity. We compared the two groups to avoid the possibility of an environmental bias. In fact, if paired with healthy control subjects, as done by present studies [12], any cognitive deficit found in BMD patients could possibly be related to social and psychological difficulties encountered by BMD children in developmental age due to their motor disease and general frailty.

The results show that BMD subjects have a preserved global intellectual functioning compared to LGMD patients, with a non-significantly decreased score at the Raven Coloured Progressive Matrices Test, assaying logical reasoning. This is in line with the existing literature, according to which average QI is normal or borderline compared to normal population QI [12].

No deficits in language, memory, and praxis emerged compared to LGMD patients, but the results showed a selective deficit in executive functions (see Table 3). Within EF, BMD patients seem to fail at complex tasks requiring sustained attention and the use of working memory, as is shown by their lower performance at PASAT and significantly reduced scores at Dual Task. Having normative data available, we also compared the frequencies of pathological scores in the two groups (see Table 3). At the Dual Task and at PASAT, pathological scores are significantly more frequent in the BMD group, whereas all LGMDs report normal scores. In other tests, assaying selective attention and working memory, such as the Trail Making Test and Alternate Fluency Test, BMD and LGMD patients performed comparably. These two tests also require WM and cognitive flexibility, since they ask to alternate tasks that require different thinking strategies, but PASAT and Dual Task entail a higher commitment of WM for prolonged periods of time. It would appear, therefore, that BMD patients are able to automate simple tasks and possibly to modulate them, but that they encounter significant difficulties in automating conscious tasks requiring high attentional engagement throughout their duration.

Of note is the wide variability in the results at the Dual Task and PASAT (DS Dual Task 21.45, DS PASAT 11.94). A wide range in the scores has also been observed in studies investigating global intelligence [12] and BMD neuropsychological profile [14]. It could depend on the different degrees of expression of dystrophin and particularly of its brain isoforms.

A further consideration is the fact that the scores obtained by BMD and LGMD patients on the Dual Task and the Drawing Copy Test were not normally distributed, nor were the scores obtained by BMD patients on the Corsi Span-Backward and FAB. As these are tasks that require motor skills, it is possible that the motor severity of both BMD and LGMD patients influences the distribution of the scores, but not the comparison between the groups.

In agreement with our hypotheses, the neuropsychological profile of BMD subjects seems to be characterized by a selective deficit of Working Memory, the executive system that deals with the manipulation of temporarily stored information for the performance of cognitive tasks. This is in accordance with the emerging literature [13, 14] that until now has preliminary assessed WM through the WM index of the Weschler Scale of Intelligence. This deficit in WM could be ascribed to the metabolic burden given by the presence of abnormal dystrophin. On the one hand, lack of dystrophin may result in the limitation of cellular processes not strictly necessary for survival (e.g., neuromediator synthesis and its transmission to the synaptic button), given its importance in cellular energy and metabolism; on the other hand, it could lead to intrinsic membrane instability, which interferes with the transmission of electrical impulses. The result is an impaired neuronal communication, affecting prominently the dorsolateral prefrontal cortex DLPFC, and the conscious processes underlying cognitive functions that require a prolonged state of cortical activation, such as WM.

We believe it would be of great interest to perform fMRI studies of DLPFC as well as of the circuitry that serves as the anatomical substrate for working memory functions in BMD subjects. A pilot study by Biagi et al. [14] in 2022 has been the first to confront neuropsychological profiles in BMD and DMD and brain connectivity through fMRI. Interestingly enough, in BMD children, there is a correlation between a reduced WM index at WAIS and a reduced connectivity between the prefrontal cortex and cerebellum (cortico-ponto-thalamic tract), both known anatomical substrates for WM.

We would like to draw attention to some criticisms of our study. Given the low incidence of the diseases, we could not pair LGMD control subjects with BMD patients according to clinic and demographic characteristics. Even though the average disease severity between the two groups does not differ, five LGMD patients were accused of high upper arms or bucco-facial severity that prevented them from completing some of the tasks, while no BMD patients exhibited motor deficits that hindered task execution. Furthermore, although BMD occurs only in males, we decided to include female subjects in the LGMD controls, in order not to limit the sample size. However, this increases the risk of error in the comparison, even though the analyses were performed on scores corrected for demographic variables as normative data were available.

The selection of the control group deserves further comment. We excluded from our study LGMD patients with *LARGE*, *POMTGnT1*, *POMTT1*, and *POMTT2* mutations, which are all related to α-glycotransferase disfunction and are associated in the literature with brain malformations with cognitive impairment and epilepsy [38]. Nonetheless, other LGMDs could potentially have an altered neuropsychological profile, despite the absence of structural brain abnormalities or even with an average normal QI and preserved global brain function. This has been reported for another α-glycotransferase-related LGMD, that is R9-FKRP or LGMD2I [39], and it could be true in R3-R4-R5-R6 LGMDs, which are related to sarcoglycans deficiency, even though these subjects are as of today considered cognitively normal. Both dystroglicans and sarcoglycans are in fact functionally related to dystrophin and are expressed in the brain, although at lower levels than dystrophin isoforms [40]. In our cohort, we have one patient affected by LGMD R9, FKRP-related, and he did not fall at any test of the battery. Despite being rare diseases, we also have 6 out of 19 patients affected by LGMD R3, R4, and R5. None of our LGMD patients showed signs of global cognitive impairment, and we did not attempt to exclude LGMD patients with selective deficits in cognitive functions. Evaluating the neuropsychological profile of R3 to R6 LGMDs in search of an executive functions deficiency, possibly mirroring the result of lack of dystrophin, could be an interesting object of further research. As of this study, the significant difference in WM function emerging from the two groups emphasizes even more so the weight of dystrophin deficiency on prefrontal functions.

Finally, in order to focus on higher-level functions, we excluded from the study BMD patients suffering from neuropsychiatric disorders, despite the high prevalence in BMD clinical spectrum of comorbidities such as autism spectrum disorders, ADHD, schizophrenia, and intellectual disability. These conditions represent the most severe consequences of partial dystrophin deficiency; therefore, the exclusion of these patients suggests a higher prevalence of cognitive disturbances in the BMD population than found in this study [12]. We believe that neuropsychiatric disorders in BMD are likely associated with a prefrontal disfunction that does not limit to DLPFC but involves the orbitomesial cortex, causing alterations in the limbic system responsible for clinical pictures of varying severity. This hypothesis too could be the object of further research.

In conclusion, patients with Becker’s muscular dystrophy fall extremely selectively on complex tasks requiring the use of working memory.

Understanding the neuropsychological profile and cognitive and neuropsychiatric comorbidities of BMD subjects could help create specific and sensitive assessment protocols, to be offered early at the time of diagnosis, as is already standard of care for DMD patients [41]. On the other hand, the neuropsychological study of patients with dystrophinopathies can help us better understand what anatomical substrates and physiological mechanisms underlie complex cognitive functions such as executive functions and working memory.

## Data Availability

Datasets cannot be made publicly available on both ethical and legal grounds, but may be made available upon reasonable request of interested researchers to the Corresponding Author, who will in turn forward a request for a data transfer agreement to the relevant Ethical Committee.

## References

[1] Ricotti V, Roberts RG, Muntoni F (2011). Dystrophin and the brain. Dev Med Child Neurol.

[2] Chamova T, Guergueltcheva V, Raycheva M, Todorov T, Genova J, Bichev S, Bojinova V, Mitev V, Tournev I, Todorova A (2013). Association between loss of function of Dp140 and cognitive impairment in Duchenne and Becker dystrophies. BJMG.

[3] Aranmolate A, Tse N, Colognato H (2017). Myelination is delayed during postnatal brain development in the mdx mouse model of Duchenne muscular dystrophy. BMC Neurosci.

[4] Rae M, O’Malley D (2016). Cognitive dysfunction in Duchenne muscular dystrophy: a possible role for neuromodulatory immune molecules. J Neurophysiol.

[5] García-Cruz C, Merino-Jiménez C, Ceja V, Aragón J, Siqueiros-Márquez L, Reyes-Grajeda JP, Montañez C (2019). The dystrophin isoform Dp71eΔ71 is involved in neurite outgrowth and neuronal differentiation of PC12 cells. J Proteomics.

[6] Lee JS, Pfund Z, Juhász C, Behen MF, Muzik O, Chugani DC, Nigro MA, Chugani HT (2002). Altered regional brain glucose metabolism in Duchenne muscular dystrophy: a pet study. Muscle Nerve.

[7] Taylor PJ, Betts GA, Maroulis S, Gilissen C, Pedersen RL, Mowat DR, Johnston HM, Buckley MF (2010). Dystrophin gene mutation location and the risk of cognitive impairment in Duchenne muscular dystrophy. PLoS One.

[8] Doorenweerd N, Dumas EM, Ghariq E, Schmid S, Straathof CS, Roest AA, Wokke BH, van Zwet EW, Webb AG, Hendriksen JG, van Buchem MA, Verschuuren JJ, Asllani I, Niks EH, van Osch MJ, Kan HE (2017). Decreased cerebral perfusion in Duchenne muscular dystrophy patients. Neuromuscul Disord.

[9] Cotton S, Voudouris NJ, Greenwood KM (2001). Intelligence and Duchenne muscular dystrophy: full-scale, verbal, and performance intelligence quotients. Dev Med Child Neurol.

[10] D'Angelo MG, Lorusso ML, Civati F, Comi GP, Magri F, Del Bo R, Guglieri M, Molteni M, Turconi AC, Bresolin N (2011). Neurocognitive profiles in Duchenne muscular dystrophy and gene mutation site. Pediatr Neurol.

[11] Ricotti V, Mandy WP, Scoto M, Pane M, Deconinck N, Messina S, Mercuri E, Skuse DH, Muntoni F (2016). Neurodevelopmental, emotional, and behavioural problems in Duchenne muscular dystrophy in relation to underlying dystrophin gene mutations. Dev Med Child Neurol.

[12] Ferrero A, Rossi M (2022). Cognitive profile and neuropsychiatric disorders in Becker muscular dystrophy: a systematic review of literature. Neurosci Biobehav Rev.

[13] Young HK, Barton BA, Waisbren S, Portales Dale L, Ryan MM, Webster RI, North KN (2008). Cognitive and psychological profile of males with Becker muscular dystrophy. J Child Neurol.

[14] Biagi L, Lenzi S, Cipriano E, Fiori S, Bosco P, Cristofani P, Astrea G (2021). Neural substrates of neuropsychological profiles in dystrophinopaties: a pilot study of diffusion tractography in imaging. Plos One.

[15] Miller EK (2000). The prefrontal cortex and cognitive control. Nat Rev Neurosci.

[16] Ericsson KA, Kintsch W (1995). Long-term working memory. Psychol Rev.

[17] Lemire-Rodger S, Lam J, Viviano JD, Stevens WD, Spreng RN, Turner GR (2019). Inhibit, switch, and update: a within-subject fMRI investigation of executive control. Neuropsychologia.

[18] Yizhar O (2012). Optogenetic insights into social behavior function. Biol Psychiatry.

[19] Salehinejad MA, Ghanavati E, Rashid MHA, Nitsche MA (2021). Hot and cold executive functions in the brain: a prefrontal-cingular network. Brain Neurosci Adv.

[20] Sakagami M, Pan X (2007). Functional role of the ventrolateral prefrontal cortex in decision making. Curr Opin Neurobiol.

[21] Padoa-Schioppa C, Conen KE (2017). Orbitofrontal cortex: a neural circuit for economic decisions. Neuron.

[22] Badre D, D'Esposito M (2009). Is the rostro-caudal axis of the frontal lobe hierarchical?. Nat Rev Neurosci.

[23] Niedman TA, Laird AR, Ray KL, Dean YM, Glahn DC, Carter CS (2012). Meta-analytic evidence for a superordinate cognitive control network subserving diverse executive functions. Cogn Affect Behav Neurosci.

[24] Godfrey C, Clement E, Mein R, Brockington M, Smith J, Talim B, Straub V, Robb S, Quinlivan R, Feng L, Jimenez-Mallebrera C, Mercuri E, Manzur AY, Kinali M, Torelli S, Brown SC, Sewry CA, Bushby K, Topaloglu H, North K, Abbs S, Muntoni F (2007). Refining genotype phenotype correlations in muscular dystrophies with defective glycosylation of dystroglycan. Brain.

[25] Bérard C, Payan C, Hodgkinson I, Fermanian J, MFM Collaborative Study Group (2005) A motor function measure for neuromuscular diseases. Construction and validation study. Neuromuscul Disord 15(7):463–470. 10.1016/j.nmd.2005.03.004 10.1016/j.nmd.2005.03.004 16106528

[26] Baddeley A (1996). The fractionation of working memory. Proc Natl Acad Sci USA.

[27] Saetti MC, Difonzo T, Sirtori AM, Negri L, Zago S, Rassiga C (2021). The paced auditory serial addition task (PASAT): normative data for the Italian population. Neuropsychol Trends.

[28] Monaco M, Costa A, Caltagirone C, Carlesimo GA (2013). Forward and backward span for verbal and visuo-spatial data: standardization and normative data from an Italian adult population. Neurol Sci..

[29] Appollonio I, Leone M, Isella V, Piamarta F, Consoli T, Villa ML, Forapani E, Russo A, Nichelli P (2005). The Frontal Assessment Battery (FAB): normative values in an Italian population sample. Neurol Sci.

[30] Basso A, Capitani E, Laiacona M (1987). Raven’s coloured progressive matrices: normative values on 305 adult normal controls. Funct Neurol.

[31] Giovagnoli AR, Del Pesce M, Mascheroni S, Simoncelli M, Laiacona M, Capitani E (1996). Trail making test: normative values from 287 normal adult controls. Ital J Neurol Sci.

[32] Costa A, Bagoj E, Monaco M, Zabberoni S, De Rosa S, Papantonio AM, Mundi C, Caltagirone C, Carlesimo GA (2014). Standardization and normative data obtained in the Italian population for a new verbal fluency instrument, the phonemic/semantic alternate fluency test. Neurol Sci.

[33] Novelli G, Papagno C, Capitani E, Laiacona M, Cappa SF, Vallar G (1986). Tre test clinici di memoria verbale a lungo termine: Taratura su soggetti normali. Arch Psicol Neurol Psichiatr.

[34] Bertolani L, De Renzi E, Faglioni P (1993). Test di memoria non verbale di impiego diagnostico in clinica: taratura su soggetti normali. Arch Psicol Neurol Psichiatr.

[35] Kaplan E, Goodglass H, Weintraub S (1983). Boston naming test.

[36] Spinnler H, Tognoni G (1987). Standardizzazione e taratura Italiana di test neuropsicologici. Ital J Neurol Sci.

[37] Beck AT, Steer RA, Brown GK (1996). BDI-II: Beck depression inventory manual.

[38] Clement EM, Godfrey C, Tan J, Brockington M, Torelli S, Feng L, Brown SC, Jimenez-Mallebrera C, Sewry CA, Longman C, Mein R, Abbs S, Vajsar J, Schachter H, Muntoni F (2008). Mild POMGnT1 mutations underlie a novel limb-girdle muscular dystrophy variant. Arch Neurol.

[39] Palmieri A, Manara R, Bello L, Mento G, Lazzarini L, Borsato C, Bortolussi L, Angelini C, Pegoraro E (2011). Cognitive profile and MRI findings in limb-girdle muscular dystrophy 2I. J Neurol.

[40] Barton ER, Pacak CA, Stoppel WL, Kang PB (2020). The ties that bind: functional clusters in limb-girdle muscular dystrophy. Skelet Muscle.

[41] Birnkrant DJ, Bushby K, Bann CM, Apkon SD, Blackwell A, Colvin MK, Cripe L, Herron AR, Kennedy A, Kinnett K, Naprawa J, Noritz G, Poysky J, Street N, Trout CJ, Weber DR, Ward LM, DMD Care Considerations Working Group (2018) Diagnosis and management of Duchenne muscular dystrophy, part 3: primary care, emergency management, psychosocial care, and transitions of care across the lifespan. Lancet Neurol 17(5):445–455. 10.1016/S1474-4422(18)30026-7 10.1016/S1474-4422(18)30026-7 PMC5902408 29398641

